# Episodic curiosity for avoiding asteroids: Per-trial information gain for choice outcomes drive information seeking

**DOI:** 10.1038/s41598-019-47671-x

**Published:** 2019-08-02

**Authors:** Linus Holm, Gustaf Wadenholt, Paul Schrater

**Affiliations:** 1 Department of Psychology, Umeå University, S-901 87 Umeå, Sweden; 2 Department of Psychology, University of Minnesota, Minneapolis, MN 55455 USA; 3 Department of Computer & Engineering Science, University of Minnesota, Minneapolis, MN 55455 USA

**Keywords:** Human behaviour, Computer science

## Abstract

Humans often appear to desire information for its own sake, but it is presently unclear what drives this desire. The important role that resolving uncertainty plays in stimulating information seeking has suggested a tight coupling between the intrinsic motivation to gather information and performance gains, construed as a drive for long-term learning. Using an asteroid-avoidance game that allows us to study learning and information seeking at an experimental time-scale, we show that the incentive for information-seeking can be separated from a long-term learning outcome, with information-seeking best predicted by per-trial outcome uncertainty. Specifically, participants were more willing to take time penalties to receive feedback on trials with increasing uncertainty in the outcome of their choices. We found strong group and individual level support for a linear relationship between feedback request rate and information gain as determined by per-trial outcome uncertainty. This information better reflects filling in the gaps of the episodic record of choice outcomes than long-term skill acquisition or assessment. Our results suggest that this easy to compute quantity can drive information-seeking, potentially allowing simple organisms to intelligently gather information for a diverse episodic record of the environment without having to anticipate the impact on future performance.

## Introduction

Humans often gather information for reasons that seem far detached from satisfying life sustaining needs. We may leisurely browse the web, binge read a book through the night or gossip with friends for reasons best described as satisfying curiosities. Clearly, satisfying curiosities is rewarding, but what instills curious behavior is less certain. The purpose of this study was to test what drives the intrinsic motivation for information.

Since at least the 1950’s, researchers have noticed that primates seek information and interact with toys for no other apparent reason than the pleasure inherent in the activity^1,2^. Similarly, Csikszentmihalyi^3^ pointed out that many human activities are open-ended and that they capture interest based on emergent rewards intrinsic to the activity, as when children start playing with building blocks and discover new possibilities from their interactions with the toys, rather than from an explicit building goal determined from the outset. Other researchers have focused on the social context in which apparently intrinsic motivation is displayed (see e.g.^4^ for an accessible review). While these and other researchers have made great efforts at describing the phenomenon of intrinsic motivation, they have not clarified the mechanisms of intrinsic motivation. Specifically, there are substantial gaps in our knowledge about what drives intrinsic motivation for information and thus how to quantify and predict it.

The main view in the field is that learning bestows an internal reward, which incentivizes information seeking for its own sake^5–8^. Learning typically refers to long lasting changes in a system, often in the service of reducing future prediction errors. Such learning is expressed in the acquisition of skill. But learning may also refer to updating temporary knowledge states such as in learning the present weather from a casual glance out of the window or closing an episodic gap by watching the next TV series episode, and we denote this episodic learning. The question arise what signal might drive intrinsically motivated information seeking - whether for skill improvements or for completing an episodic record. It seems an initial state of uncertainty is required in both cases, or information seeking would by definition not bring about any information.

A host of studies support the view that reducing uncertainty is rewarding. Starved and water deprived rats tend to explore new environments before they eat or drink^9,10^, explore even if penalized with electric shocks^11^ and prefer to move into complex mazes over simple and predictable ones^12^, which suggest that reducing uncertainty is intrinsically rewarding, and that information seeking may compete with actions that satisfy other needs. Consistently, Katz and Gelbart^13^ showed that opiate receptor blockades reduced the environmental exploration rate of rats. Furthermore, dopamine antagonists substantially reduced the preference for environments associated with novel objects in rats^14^. Moreover, the lateral habenula neurons of macaque monkeys respond to information prediction errors in the same way as reward prediction errors^15^. In humans, resolving perceptual ambiguity activates striatum^16^ and anticipation for trivia question answers activates the caudate^6^ which are brain regions that respond to reward and the expectation for reward, respectively. Moreover, human choice behavior suggest information itself is valued in exploration-exploitation trade-offs^17^ and possibly proportional to the uncertainty of exploration yielding a positive *explicit* reward prediction error^18^ and expected horizon for exploration^18,19^. In addition, uncertainty is often strongly aversive^20^, and the unease of not knowing may compel information-seeking. For instance, people prefer to know whether and when they will receive an electric shock^21^ and prefer less ambiguity in betting games, despite identical chances of winning^22,23^. Furthermore, deciding between two good outcomes induces distress inversely proportional to the value difference^24^.

Uncertainty indicates limits in the ability to predict and verify. Reducing uncertainty then, comes with a general survival value to the organism - it may improve prediction, planning and the expected value that can be achieved by an action. It has been repeatedly noted that uncertainty directs animals to explore novel content^12^; see^25^ for a review), and exploration is critical for both learning an environment, and for tracking changes that may occur both in the animal’s external environment and in the animal itself - it’s capacity, knowledge, abilities, and performance. To explore effectively, an organism needs to acquire additional appropriate information, typically through an active search and sampling of the environment termed *information-seeking*.

While uncertainty reduction may be both intrinsically and extrinsically rewarding, exploratory actions are not free - they come with costs in the form of lost opportunities and delays. To balance costs and benefits of information-seeking, the overall value of exploration is something an organism will need to evaluate. A core component of this calculation is to estimate the expected impact of information seeking on uncertainty, an idea we will formalize as *information gain*. If organisms use information gain to drive information-seeking actions, we expect that sampling behavior will increase monotonically with information gain for fixed costs and benefits.

To better understand information gain, it is helpful to view it as an information gap measured by how much uncertainty will decrease if one were to use some available sample information. This requires the agent to know how to collect the information as well as an understanding for what the information might be, but not what the actual information content is. It also requires some ability to generate an expected change in uncertainty by collecting the information. In general, information gain is highest when there is large uncertainty (a large number of potential outcomes) and high quality information available that can dispel this uncertainty. However, information gain calculations require some care to execute correctly - there may be multiple factors that influence an outcome, and using this information may require distinguishing between these factors to reduce uncertainty. For example, after years of neglect in riding a bike, untracked changes in your performance creates uncertainty about your current riding abilities. The information gain is how much of that uncertainty you can dispel by trying it out. But identifying your bike riding competence from your attempt requires the ability to separate errors due to problem difficulty from errors due to skill. Trying to ride a defective bicycle will do little to help re-calibrate your skill level. Factoring off difficulty from skill means that information gain calculations will often require credit assignment. Interestingly, for many domains, we seem able to sense difficulty from some initial perception. Beyond calibrating our current skills, information gain can also reflect an anticipation of *learning -* future skills and knowledge acquired through the sampled information. Taken together, outcome observations – or feedback – may simultaneously serve to reduce performance uncertainty, improve ongoing performance, promote learning across trials and help calibrate metacognitive understanding of skill level in the domain.

Oudeyer, Gottlieb and Lopes^8,26^ recently suggested that intrinsic motivation is dependent on learning progress (see also^27^ for a similar idea). According to the learning progress account, uncertainty may induce information seeking by signaling learning opportunity. When uncertainty remains unchanged across multiple attempts at information seeking, learning progress provides no guidance for behavior. Learning in this context therefore refers to improvements *across* trials or attempts and thus concerns learning directed towards skill improvement or acquisition of generalizable knowledge. We denote this type of learning “parameter learning” as regardless of whether the learning concerns skill improvement or acquiring a more accurate understanding for one’s own performance, it entails updating parameters representing a compressed generalizable representation via information seeking. Importantly, in parameter-learning as construed here, the change in the state of the knowledge variable is tracked and the expectation for posterior gain in information from information seeking actions is what incentivizes information seeking.

But intrinsic motivation for information may not be limited to skill improvements. As there are multiple memory systems (see e.g.^28^ for a brief review), there are also potentially multiple learning types. For instance, in addition to learning progress, there appears to be intrinsic value in simply knowing what happened next, without anticipation for skill or general knowledge improvement. We call this type of intrinsic motivation *episodic curiosity*, which incentivizes closing an immediate episodic gap in observations. The only signal required to trigger such curiosity is the uncertainty about what happened next. Possibly, bridging episodic gaps might eventually yield skill learning or generalizable knowledge, but such an outcome is not required for promoting information seeking. Thus, the episodic curiosity view complements the prediction from the learning progress account in that a reduction in prediction error across multiple information samples is not necessary for intrinsically motivated information seeking. For instance, learning how one performed on the last trial does not imply improving performance - or even identifying ones skill - but may still be sufficiently desired to induce information seeking. Such an episodic policy for information seeking may also add the advantage of producing a pool of outcomes that can be reinterpreted in the future if needed.

Our new contribution resides in explicitly distinguishing between different computations sub-served by distinct types of learning, and testing how they incentivize information seeking. Our episodic curiosity idea is similar to the knowledge gap account of curiosity^6,29^ according to which curiosity is based on a discrepancy between the current knowledge state and an aspired-for knowledge state. However, our account is significantly more general, requiring only the ability to predict or forecast outcomes without the need for knowledge state targets or explicit gap measures. While knowledge gaps are intuitive, they are challenging to quantify, and we are not aware of any previous study that directly quantifies knowledge gaps to test the theory.

Yet another possibility is that humans seek information based on an ongoing tally of outcome observations. Information seeking is then feedback-dependent. For instance, a run of several observed successful attempts would reduce the error signal and the probability of making an observation (i.e., seek information) because the outcome uncertainty is (temporally) reduced. This idea is common in reinforcement learning and the signal that drives information seeking then is typically a moving average on outcomes. Importantly, such an account does not assume the ability to sense the uncertainty from some perception, but only relies on its recent history of success and failure. Due to forgetting, this policy might then make similar predictions to our episodic curiosity prediction, but is constrained to be feedback related.

**Figure 1:**
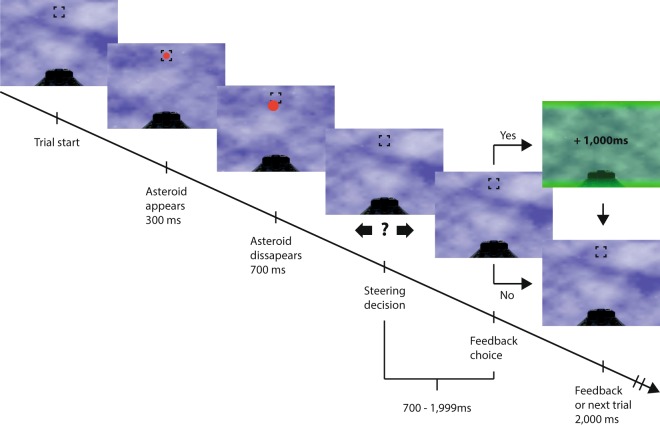
Schematic trial flowchart. Participants were given a two alternative forced choice visual discrimination task, presented as a simple computer game with the objective of steering a spaceship away from approaching asteroids, which expanded in size over time. After watching the asteroid appear and travel towards the cockpit for 400 ms, the asteroid disappeared and participants indicated where they wanted to steer the ship (left or right) to avoid collision, and subsequently, whether to request feedback in the form of steering decision outcome. Both responses were made within a 1300 ms deadline. Asteroid size increased for illustrative purposes here.

We tested the relationship between information gain and information seeking using a paradigm where we carefully measured learning and the incentive strength of information in a decision task (Fig. 1) rigged as an asteroid avoidance game. After each steering decision, performance feedback information could be sought at the cost of a time penalty. This pits the intrinsic value of performance information against the averseness of waiting^30–34^.

Two independent key principles may drive participants to seek information in terms of performance feedback in the task. First, participants may request feedback in order to learn across trials. This type of learning might include anything from performance improvement, assessment of skill, assessment of difficulty as well as changes in performance and difficulty. For performance learning, we would then expect to see feedback related improvements. For both performance improvement and skill assessment, information seeking should be higher at more difficult trials because such trials convey more information about the respective parameters. The same goes for information seeking that targets changes in those variables. Common to these instances of across-trial learning is that expected information gain monotonically decrease across trials with information requested due to the time-penalty incurred by requesting feedback. We then expect information seeking to decrease across trials. There is a possibility that the information received is not stored efficiently, producing forgetting. With forgetting, the expected information should remain high through the test. However, even under such volatile conditions, right after feedback has been requested, a new feedback request should be less likely. The second key principle for information seeking is that information is sought on a per-trial basis and driven by trial outcome uncertainty. We call this principle *episodic curiosity* and we used participants’ individual average performance at each difficulty level as proxies for outcome uncertainty. This allowed us to express participants information gain in terms of bits computed from the proportion of correct steering decisions, at each difficulty level, respectively. Moreover, if elimination of per-trial uncertainty is sufficient to drive information seeking via feedback requests, then we would *not* expect to see any change in feedback request rate. Taken together, our experimental paradigm tests whether two different learning categories (parameter or episodic learning) predict intrinsically motivated information seeking.

## Results

Decisions were implemented as an asteroid avoidance video game, illustrated in Fig. 1, where participants watched appearing asteroids and made left and right decisions to avoid collision. A set of different color-coded asteroid trajectories were used to vary decision difficulty and performance could be directly computed for each trajectory. Toward the end of each trial, participants could request avoidance outcome feedback in exchange for a time penalty. The repeated nature of the task allows for improvement across trials. The experiment thus also allowed for investigating whether information requests were related to parameter learning such as performance improvement across trials or feedback requests, as well as identification of skill via sampling to reduce posterior uncertainty about performance distribution across the trajectories.

**Figure 2:**
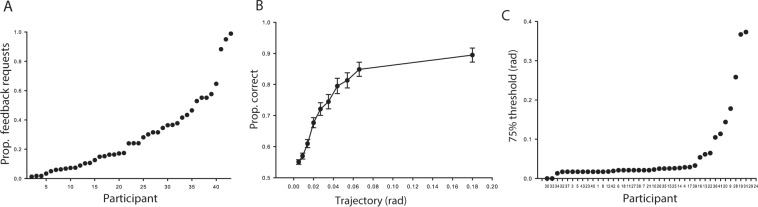
Feedback request rates and performance. Panel (A) Proportion feedback choices across participants (participants sorted on proportion feedback requested). Panel (B) Average aggregate (across participants) performance for each trajectory. Panel (C) Participants’ 75% thresholds, participant id reflect increment in feedback requests (as in Panel A).

To reliably identify a relationship between learning and feedback choice, performance and proportions of feedback choices need to be sufficiently widely distributed. As seen in Fig. 2A, no participant chose to forgo feedback completely. Furthermore, as seen in Fig. 2B, performance varied as a function of asteroid trajectory. Specifically, asteroid avoidance performance monotonically improved with asteroid off-set angle from the central meridian. Moreover, as seen in Fig. 2C, there was substantial variation in performance across participants. Thirty-three participants (77%) indicated a 75% discrimination threshold of 0.1 rad or less.

### Parameter learning and feedback request change

To test if participants choose feedback to improve parameters, we investigated time series effects in the feedback and performance data. Regardless of whether parameter learning came in the form of feedback performance improvement, assessment of improvement, assessment of skill or any other type of across-trial learning, there should be time series effects on feedback requests.

**Figure 3:**
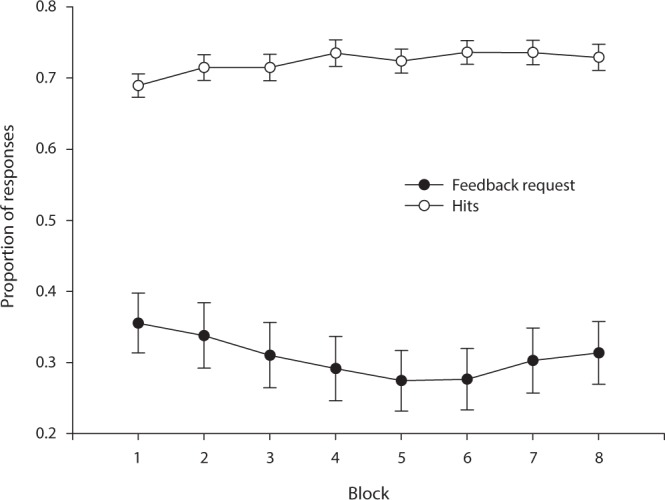
Average performance and proportion feedback requests as a function of trial block. Each block is 100 trials. Error bars are 95% CI.

First, we computed the performance means of each block of trials. As seen in Fig. 3, there is an increase in performance which then appears to stabilize around block 4. This observation was qualified by an 8(Block) repeated measures ANOVA producing *F*(7) = 4.85, *p* < 0.001 (greenhouse geisser corrected), *η*^2^ = 0.103. Moreover, contrast tests suggested the effect largely resided in a difference between the first block and the last few blocks. For instance, the contrast test between the first and the last block was reliable at *F*(1) = 9.55, *p* < 0.001 (greenhouse-geisser corrected). The same analysis was carried out with respect to feedback choices across blocks and are summarized in Fig. 3. However, there was no reliable difference in feedback choice rates between blocks, *F*(7) = 2.25, *p* < 0.09 (greenhouse-geisser corrected), *η*^2^ = 0.0501.

While performance improved slightly across the test, learning may also be evident in faster steering responses across trials. On average, participants completed their steering responses within 0.56 s. There was no reliable difference in response times between blocks of trials, *F*(7) = 1.875, *p* < 0.107 (greenhouse-geisser corrected), *η*^2^ = 0.043. Numerically, participants made about 15 ms faster steering decisions in the first 100 trials compared to the rest of the blocks, suggesting if anything that participants traded in response time for steering accuracy later on in the session, leading to constant efficiency in terms of performance per unit decision time. However, feedback request strategies may be concealed by this crude aggregation. It is still possible that performance improvements are tied to feedback requests and that the distribution of feedback requests vary with difficulty and across number of trials.

**Figure 4:**
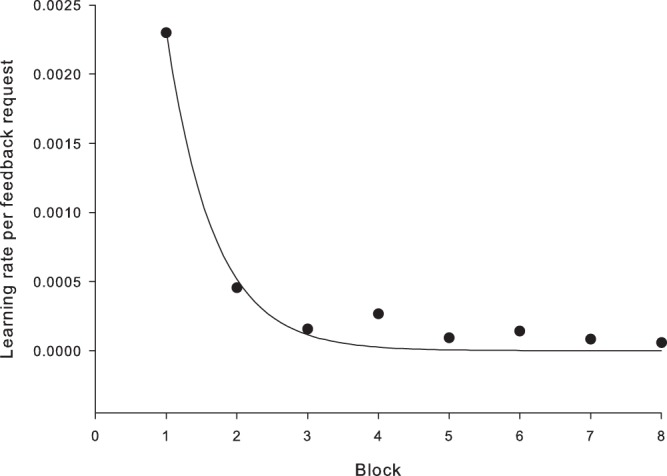
Learning rate per trial and feedback request, averaged for each trial block. Units on y-axis are proportion of performance improvement per trial. Fitted line is an exponential decay function.

As aggregate results suggested reliable learning, this opened up the possibility that participants may have requested feedback to also trace or increase their performance. If so, we should see a relationship between performance improvement and feedback requests. We aggregate across difficulty and visualize the relationship by computing the derivative of performance across participants, as a function of trial. We then aggregated the trials in the eight experimental blocks to arrive at a quantity reflecting aggregate performance improvement rate. We then divided the performance improvement rate with the proportion of feedback requests to assess the feedback related learning. As seen in Fig. 4, there is a sharp decrease in feedback related learning from the first to the second block, and the learning is essentially saturated at block three, if not already by block 2.

**Figure 5:**
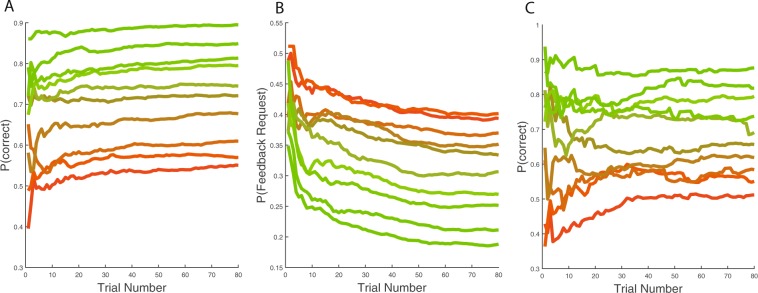
Objective and feedback-derived performance across trials. Panel A: Performance by trial and difficulty. There is initial improvement which stabilize between trial 10:20. Panel B: Feedback sampling rate by trial and difficulty (trajectory). As seen, there is initial performance related sampling suggestive of parameter learning, but it asymptotes differently depending on trajectory difficulty rather than at zero for all difficulties. Panel C: Performance estimate given feedback requests by trial and difficulty. Difficulty is reflected in line color, red is the most difficult.

There is a possibility that the aggregate results hides interactions between feedback related learning and difficulty levels (i.e., trajectory). To check for that possibility we break down the performance and feedback request results in Fig. 5. Figure 5A summarizes performance as a function of trial for each of the ten difficulty levels. The figure suggests a more fine-grained learning happening within the first block (first 10 trials in the figure). Moreover, Fig. 5B shows how sampling varies with trials for each difficulty level. It appears as if feedback requests are tied to performance improvements. Notice also that average performance as determined solely by feedback requests (Fig. 5C) appear fairly close to objective performance.

Figure 5 indicates that sampling follows a power law, which is equivalent to a mean of several exponentials with slightly different parameters. This suggests that subjects optimally collect information to set parameters. However, there is no *interaction* in performance or sampling across trials which was predicted by the parameter learning account: the decrease in sampling and increase in performance is constant across difficulty, and sampling asymptote at different levels depending on difficulty.

Another possibility in the present study is that subjects seek information based on the valence of feedback rather than based on difficulty. In general, subjects should then request less feedback, the more difficult the trial. But we find overwhelming evidence for the opposite, namely that feedback is more frequently requested the more likely the subjects are to be wrong, because subjects sample more frequently, the more difficult the trial is (see Fig. 5B).

With limited memory, the information gain for learning skill level or change in skill should remain high across the test. Participants should then continue to request feedback through the test. However, even with limited memory we would expect a reduction in information gain directly after having requested feedback. Participants should then be less likely to request feedback on trials right after they requested feedback.

**Figure 6:**
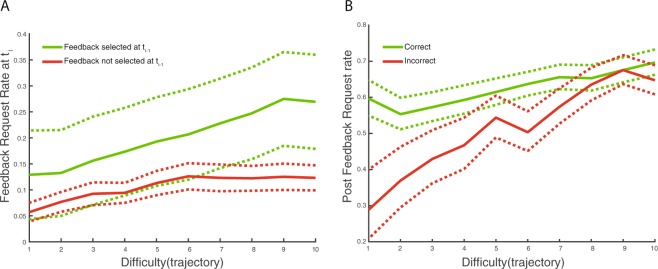
Feedback request rates given previous trial feedback choice. Panel A: Feedback request rate on trial t_i_ given feedback requested at trial t_i-1_ (green) and feedback not selected at t_i-1_ (red) as a function of difficulty. Panel B: Probability of requesting feedback following correct or incorrect feedback outcome on previous trial across difficulty. Error zones (dotted lines) indicate 95% CI.

As seen in Fig. 6A, feedback is about twice as likely to be requested if it was requested on the previous trial at that difficulty level. This pattern is directly opposite to what would be predicted had participants sought information for assessing skill level, even with forgetting. Yet another possible kind of parameter learning would be to track potential for improvement. This should bias sampling following positive feedback at difficult trajectories. But as seen in Fig. 6B, this prediction is not supported in our data. Instead, the likelihood of immediately requesting feedback following positive feedback is flat across difficulty. There is a tendency to not request feedback following negative outcomes at easier trajectories. However, the effect is not reliable. Moreover, the slight bias does not seem to have biased participants’ performance estimates (cf. objective performance in Fig. 5A and performance based on feedback requests in Fig. 5C).

Taken together, there is evidence for information seeking based on parameter learning roughly within the first 12% of the test trials and it is tied to performance improvement. Nonetheless, information seeking continues at the same rate through the entire test, and request rates remain stable for each difficulty level. Thus, having largely rejected parameter learning as a sufficient account for our results, the only viable explanation for the maintained information seeking rate seems to be episodic curiosity.

### Episodic curiosity as per-trial expected information gain: Group level analysis

**Figure 7:**
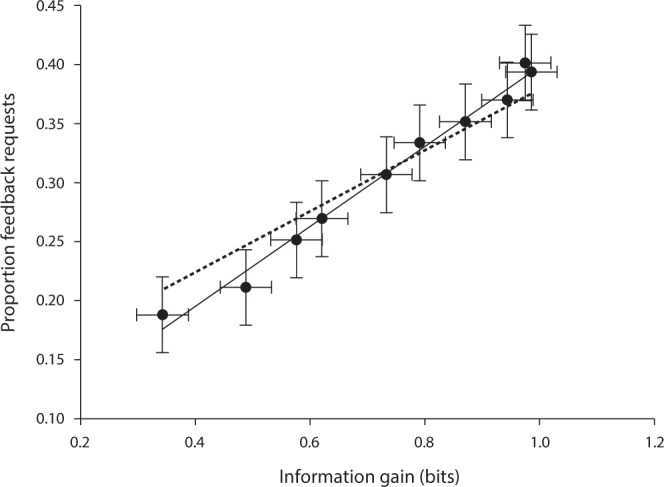
Average proportion of feedback requests as a function of information gain. Each point represents one target trajectory, averaged across all participants and trials. Error bars are 95% CI. Solid line reflects linear trend using simple linear regression for illustration purpose. Dotted line reflects the best fit linear mixed effect model, treating subject (individual differences) as a random effect. The mixed effect model effectively excludes subjects with low feedback request rates from the fit, shifting the mixed effect line upwards.

Episodic curiosity refers to the desire to close an episode and the quantity we believe drives episodic information seeking is per-trial expected information gain. For our experiment, this predicts that the proportion of feedback requests would have a simple positive relationship with increasing information gain. Having largely rejected parameter learning as a sufficient account for information seeking, and displayed that there is no interaction between difficulty, performance or information seeking, respectively, we now aggregate across trials for each difficulty level and analyze the key claim for episodic curiosity in our data. We computed information gain by taking each participants average proportion of correct steering decisions at each trajectory as a proxy for outcome uncertainty and computed the bit entropy on that proportion (i.e., the self information of a binary variable). We found an approximately linear relationship between feedback request rate and information gain, shown in Fig. 7.

To test if expected information gain predicted the proportion of feedback requests, a linear mixed-effects model was fitted with ML estimation using the *lme4* package in R^35^. The model (Equation 1) included a fixed effect (*β*_1_) for expected information gain (X_i_), and random by-subject intercepts (*S*_*0s*_) and slopes (*S*_*1s*_) for expected information gain. Following Barr, Levy, Scheepers and Tily^35^ a likelihood ratio test indicated that including the fixed effect (*information gain* + *individual differences*) resulted in a better fit compared to an (*information gain*) model without the fixed effect (Table 1). Expected information gain reliably predicted proportion of feedback requests (Table 2), with the fixed effects suggesting that subjects, on average, gained 0.12 + 0.28 bit/s invested on requesting feedback. The *β*_0_ parameter suggests an exploration rate independent of information gain. See Table 2 for R-compatible formula. Random effects are presented in Supplementary Fig. S1.

1 $$\begin{array}{rcl}{Y}_{si} & = & {\beta }_{0}+{S}_{0s}+({\beta }_{1}+{S}_{1s}){X}_{i}+{e}_{si}\\ ({S}_{0s},{S}_{1s}) &  \sim  & N(0,[\begin{array}{cc}{\tau }_{00}^{2} & \rho {\tau }_{00}\rho {\tau }_{11}\\ \rho {\tau }_{00}{\tau }_{11} & {\tau }_{11}^{2}\end{array}])\\ {e}_{si} &  \sim  & N(0,{\sigma }^{2})\end{array}$$

**Table 1:** Measures of model fit for the Information gain model.

Model	*df*	*AIC*	*BIC*	*LogLikelihood*	*Χ*^2^
Null	5	−851.21	−830.89	430.61	
Information gain	6	−872.78	−848.39	442.39	23.565***

*Notes*. *p < 0.05. **p < 0.01. ***p < 0.001.

N_subject_ = 43.

Observations = 430.

**Table 2:** Results from the linear mixed-effects analysis of Information gain on Feedback choice.

Formula	*FB*~*IG* + (*IG*|*Subject*)	
**Fixed effects**	*β* (95% CI)	*t* (df)
Intercept	0.12 (0.01–0.22)	2.11* (40.53)
Information gain	0.28 (0.19–0.37)	5.93*** (40.94)
**Random effects**		
σ^2^	0.00	
*τ*_00,Subject_	0.11	
ρ_01_	−0.50	
ICC_Subject_	0.97	

*Notes*. All *dfs* subject to Satterthwaite corrections. *p < 0.05. **p < 0.01. ***p < 0.001.

σ^2^ - within-group variance.

*τ*_00,Subject_ - between-group-variance.

ρ_01_ - random-slope-intercept-correlation.

ICC_Subject_ - intraclass correlation.

N_subject_ = 43.

Observations = 430.

To examine explained variance, conditional *R*^2^ ^36,37^ was computed using the *MuMIn* package in R^38^, *R*^2^_*GLMM(C)*_ = 0.97. The strong linear relationship suggests that information gain is the primary determinant of aggregate feedback request rate.

The above analysis is based on aggregating across trials from the entire test including the first part which contained some evidence for parameter learning. To rule out any time series effects concealed by this aggregation, we also separately analyzed each block of 100 trials with separate linear mixed effects models. The results showed a slight change in intercept but similar slopes across blocks. These results are summarized in Supplementary Fig. S2.

### Episodic curiosity as per-trial expected information gain: Individual analysis

**Figure 8:**
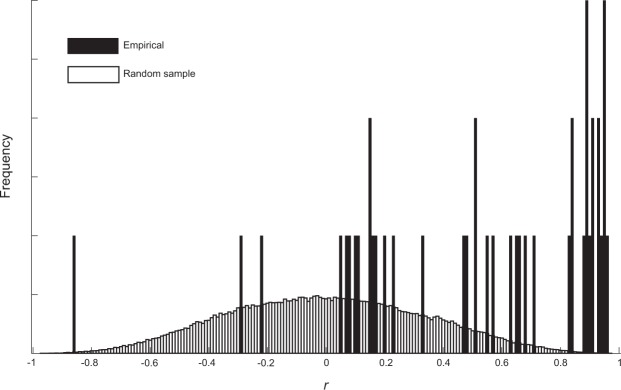
Random permuted request proportion to information gain *r*-scores are depicted in white bars. Empirical *r-*scores for feedback request to information gain in black.

While the relationship between information gain and feedback requests is strong at the aggregate level, Fig. 2 shows large individual differences in feedback request rates. Some participants have extremely high and others extremely low feedback request rates, raising the possibility that our results are driven by a small subset of subjects. We therefore computed the linear regression coefficients between information gain and feedback requests for each participant. For comparison with this association distribution, we performed a Monte Carlo permutation test to determine the null hypothesis distribution of *r* values if there was no relationship between information gain and feedback requests. Feedback requests were randomly assigned across trajectories within each participant. The average *r* value from 10000 participant and trajectory sets of simulations produced a distribution with an average *r* = 0.0, and *SD*_*r*_ = 0.33 (see Fig. 8). In contrast, the distribution of the empirical *r*-scores is substantially higher, with an empirical average *r* = 0.47, which in the simulation has a *p* < 0.0001. Based on the permutation test, *r*-values higher than 0.63 are unlikely to happen by chance (*p* < 0.05), and 24/43 participants have *r* values above this criterion.

To also get a sense of what dominates feedback request, we ran a principal component analysis on feedback request across difficulty and participant. The results summarized in Supplementary Fig. S5 show that the first component corresponding to overall feedback request rate dominates with about 98% of the variance explained. The second component captures the slope of increasing feedback request rates with difficulty. While the dominant factor in our data is individual differences on overall feedback request rate and disrupts the information gain-information seeking relationship, it pose no threat to our claim. Individual regression fits of feedback rates vs. information gain showed that the majority of subjects displayed a positive relationship (see Fig. 8). Subjects with very high and low feedback request rates were most likely to have low correlation coefficients, presumably due to ceiling (and floor) effects. However, one participant displayed a strong negative effect of expected information gain on proportion feedback choices.

## Discussion

This study tested what information quantity drives intrinsically motivated information seeking. We identified and tested two broad learning incentive categories: across-trial (parameter) learning and per-trial (episodic) learning. We received marginal support for the position that across-trial learning progress drives information seeking. After about 100 trials corresponding to the first 12% of the test trials, we found no further support for parameter learning driving information seeking whereas participants kept requesting feedback at the same rate through the test. The finding is consistent with parameter learning driving information seeking as predicted by the learning progress hypothesis, but this interpretation is not unanimous given the fact that information seeking did not diminish across the test. However, participants may have forgotten the feedback they received on prior trials and this would then lead to maintained levels of expected information gain for parameter learning. But even with forgetting, participants should have less expected information gain immediately following feedback and we found no support for this in our data. Instead, participants were more inclined to request feedback, had they requested feedback on the previous trial. Moreover, participants might have sampled differently depending on the actual outcome on the previous trial if participants sought information about change in performance. For instance, evidence of incorrect steering decisions at easy trials and correct steering decisions at difficult trials should produce *surprise* and incentivize further information seeking in order to identify skill improvement (see also e.g.^39,40^, regarding surprise and interest). But we found no evidence for that behavior in our data. Instead, we received overwhelming support for per-trial information gain driving information seeking. At the aggregate as well as individual levels, we found strong support for a linear increase in information seeking as a function of uncertainty. Furthermore, information gain kept driving information seeking across the entire test (see also Supplemental Fig. S2).

Our aggregate level finding of a monotonic increase in information seeking with increased expected information gain goes against the present knowledge gap theory of curiosity which maintains that the relationship between expected information gain and curiosity is an inverted u-function^6,29^. However, the earlier reported findings suggesting an inverted u-function of curiosity and uncertainty is also predicted under our account *if* posterior predictability at high prior uncertainty is low. This would for instance be the case if feedback could not be interpreted and therefore provide no knowledge gain (see^39,40^ for a similar but not directly tested idea). Conversely, the inverted u-function reported in earlier studies may reside in test items not providing posterior knowledge advance. For instance, Kang *et al*. used trivia questions where participants could trade off money or time against learning the answer to the trivia question. The probability of requesting a costly answer was an inverted u-function of self-rated confidence on what the answer might be. Subjective confidence is not identical to information-gain. Information-gain requires that the observer has enough knowledge to accurately compute the change in posterior knowledge *before* information is acquired. Low subjective confidence can also indicate cases where information-gain cannot be computed. Importantly, the trivia questions not well understood by a participant would also be the ones eliciting the weakest confidence. Furthermore, if the question was not well understood, then receiving its answer would provide no change in the posterior distribution (i.e, no information gain).

Our findings could not be predicted by the learning progress account of intrinsic motivation suggested by Oudeyer, Gottlieb & Lopes^16^. We found evidence for feedback related learning only across the first 100 and possibly into the 200’th trials out of 800. Indeed, there was no other evidence for across-trial changes in behavior in the data. For instance, we found no reliable evidence for a change in overall feedback request rate across trials, and no interaction between trajectory and trial with respect to feedback request, which would have been expected had participants sampled to identify their skill or skill given trajectory, respectively. Furthermore, parameter learning with forgetting does not seem a likely candidate for explaining our results either. We found no reliable effects of feedback request rates contingent on preceding feedback selection, and no reliable interaction between negative or positive feedback outcomes with difficulty levels. Thus, it appears as if intrinsically motivated information seeking does not require an expectation for parameter learning - only a sense of uncertainty regarding per-trial outcome and presumably an expectation that the information seeking action can dissolve it. We interpret our findings as reflecting an incentive to learn in the sense of closing an episode and that this incentive is sufficient to drive information seeking.

Our key finding that outcome uncertainty is sufficient to drive information seeking builds on earlier ideas linking uncertainty and curiosity^5,41^. Schmidhuber^42^ suggested predictive compressibility as a principle for information seeking in artificial agents, which is fully consonant with our main finding. However, Schmidhuber and other artificial intelligence researchers have argued that information seeking based on predictive compression can lead to rewarding unpredictable inputs (e.g.^27,43^), which may cause an agent to oversample irrelevant information. As a consequence, these researchers have shifted focus to uncertainty reduction in the parameter learning sense.

We expanded on those ideas by distinguishing between different types of learning, and formalized how those differ in how they drive intrinsic motivation for information. Moreover, our computational distinction between parameter and episodic curiosity may be related to learning in different memory systems (see e.g.^28^ for a review). Testing how different types of curiosity affects learning in different memory systems seem like an important field of future enquiry.

The simplicity of per-trial episodic uncertainty as a driver for information seeking may belie the complexity of the learning opportunities it might provide. Operating on episodic uncertainty when seeking information offers the opportunity to improve skill understanding as well as skill improvement *if* such opportunity exists. But without seeking information, there is no way to know. In principle, several possible learning opportunities may be satisfied with a single information seeking action, and a rational approach to deciding which information seeking action to take ideally involves computing the posterior distribution of information gains from all available knowledge variables and actions. But computing the posterior distribution across all of those imaginary knowledge gains seems like a rather computationally challenging endeavor. If operating on a simple cue such as per-trial uncertainty often offers similar learning opportunity as a more hypothesis-based principle, then episodic uncertainty reduction might constitute a computationally efficient principle, at least for some cases. Moreover, the simplicity may also mean that a range of animals may utilize the same or similar policies for information seeking. A caveat with an information seeking policy based on per-trial uncertainty is that one might easily get stuck and continue to explore domains without any other benefit than receiving repeated information gains about the episode. This then also forms an empirical prediction; a lottery without stakes might captivate the observer (see^44^ for some recent evidence in this direction). However, one point raised here and also explicitly outlined in our theoretical section is that episodic curiosity operates as one out of possibly several information quantities to drive information seeking. Furthermore, these different information quantities driving information seeking may differ according to learning context. For instance, in a situation where the simple uncertainty reduction computation advocated here produces substantially lower information gains than a more hypothesis-driven principle such as sampling for changes in performance or the world, one might expect more evidence for the latter principle in human information seeking behavior. Another possibility is that different information quantities operate independently, perhaps even under independent reward systems, involving different memory systems.

The asteroid avoidance game used in the present study may have induced an expectation on the participants to perform well and might therefore be conceived of as testing extrinsic motivation. For instance, participants may have been competitive and viewed positive feedback as a reward in terms of confirmation of achievement. Moreover, participants may have valued positive feedback (confirmation of successful steering action) more on the difficult trials and therefore requested feedback more frequently the more difficult the trajectory. But then such sampling would also mean participants got more exposed to negative feedback. Furthermore, we found no evidence for reliable differences in requesting feedback contingent on its valence. It is possible that participants may have felt an obligation to occasionally request information for task compliance, but not *when* they should request feedback. A potentially more relevant objection is that participants were genuinely interested in improving their skills and thus stoically took the higher rate of negative feedback at difficult trials in favor of improvement. But if that kind of motivation is construed as extrinsic motivation, it seems the term extrinsic motivation essentially loses its meaning, because what then would constitute intrinsic motivation? Furthermore, the extrinsic motivation account of our findings would have been a serious concern had participants displayed across-trial learning related feedback requests. Because we found only limited evidence for skill learning related information seeking, it seems more accurate to interpret our findings as expressions of intrinsically motivated information seeking.

In this study we do not distinguish information seeking as driven by an expectation for an information reward, from information seeking incentivized by aversion to uncertainty. Future studies may distinguish between these accounts and assess the valence involved in people’s’ information seeking choices. Regardless of whether seeking information or avoiding risk is the more appropriate explanation for the present findings, neither information reward studies nor risk aversion studies have to our knowledge explicitly derived or tested the computational principle we investigated in this study, namely that per-trial uncertainty intrinsically motivates information seeking.

A different possible outcome of the experiment would be if participants sought to preserve their beliefs against feedback (avoidance of incongruent information). Such self-confirming behavior would be expected from participants displaying preferences for positive feedback and aversion to negative feedback or confirmation bias (see^45^ for a review). In the present study, confirmation seeking behavior appear to have been limited to a single participant. Instead, most participants were more likely to request feedback, the higher the risk was of receiving negative or counter-indicative feedback. Nonetheless, information seeking and information avoidance present competing and conflicting goals that may exist in different degrees across participants, and may account for the distribution of information gain to information seeking relationships found across our participants. While we demonstrate clear effects in the aggregate as well as at the individual level in favor of per-trial information seeking driven by information gain, the main component in feedback request rate is due to variations between individuals. Future studies should investigate these individual differences further. For instance, are they accounted for by self-esteem, personality, general intelligence, learning capacity, or is there some other principled reason people become curious and invest in information seeking in a given task?

## Methods

### Participants

Forty-three participants (20 females, age 19–42, *M* = 26.1) with normal or corrected to normal eyesight were recruited through Umeå University campus advertisements and were reimbursed 99 SEK (about 12 USD). The paradigm was approved by the university ethical board (EPN: 2014/110-31Ö) and in accordance with the Helsinki declaration. Written informed consent was obtained from each participant. Assuming the tested linear regression of feedback request rate as a function of information-gain would be at least *r* = 0.2, then at alpha = 0.05, this would require a sample size of n = 33.43 based on the pwr.f2.test in R^46^. The 43 participants tested in this study should therefore be sufficient to identify such a relationship.

### Equipment and software

A height-adjustable chair and a chin- and head-rest were used to position participants at about 75 cm viewing distance from the computer screen. An EyeLink 1000 sampling at 500 Hz was used to record the participants’ eye movements. Stimuli were presented on a 532 × 299 mm LCD screen with 1920 × 1080 resolution and a pixel size of 0.27 mm. In-house developed Matlab code (R2010 32b, with Psychtoolbox^30^, was used to present stimuli and record participant inputs.

### Stimulus

The asteroids appeared in ten difficulty levels corresponding to angular deviations from the midline (0.005 to 0.180 radians), left or right of the participant’s point of view (see Fig. 1 for one example). The following function expresses the deviation from the midline (in mm) at any point in time:

2 $${d}_{x}=vt\,\tan (\gamma )$$

where *v* = 200/0.27 pixels/sec, *t* = time traveled in seconds, *γ* = stimulus trajectory angle. The asteroid expanded in size as a function of time, to give the illusion of an approaching object. This expansion happened at a rate of 200% per second, starting out at a diameter of 4.6 mm. Asteroids were color and texture coded according to difficulty, allowing participants to quickly discriminate between them. No other independent variable than asteroid trajectory was employed in the present study.

### Procedure

In each trial, a flare was displayed for 200 ms to prepare the participant for the upcoming asteroid, the expanding stimulus was presented, and then a steering decision was made. After each steering decision indicated by pressing right or left arrow key on the keyboard, participants could request feedback by fixating the cockpit for 200 ms with their eyes. Once our gaze-contingent algorithm detected a fixation in the cockpit zone (i.e., a feedback request), the optical flow paused for one second, during which auditory and visual feedback was presented indicating whether the asteroid collided or was successfully avoided. A trial could thus last 2 seconds (no feedback) or 3 seconds (feedback) producing a total test time range from 26.67 to 40 minutes.

Participants first practiced the task in the presence of the experimenter to verify task compliance. Practice ended after at least ten trials with feedback requests and ten trials without feedback were completed. On average *M* = 45.77 trials (*SD* = 22.49) were completed during the practice round. Participants then continued with 800 trials organized into eight blocks of 100 trials with short participant-determined breaks between every block. In a block of 100 trials, each asteroid trajectory appeared 10 times. Missed response deadlines returned the trial to the set of remaining trials within the block, guaranteeing 800 responses from each participant.

### Information sampling theory and active learning

The problem of deciding when to sample data to improve predictions or performance is called active learning. Active learners try to maximize their learning objective using the least number of sample data points, due to the costs of collecting information as in our experiment. We distinguish two types of active learners. Parameter learners try to maximize performance by updating uncertain parameters compiling experience across trials. Episodic learners instead seek feedback for particular outcomes, without necessarily aggregating these outcomes into parameters. Episodic learners can be considered *pool-based* active learners, in the sense that they collect a pool of outcome labeled data that can be stored and potentially mined for unrelated future tasks.

#### Information theoretic approach to analyzing information incentive for parameter learning

While active learning is typically framed in terms of optimizing a particular decision, information theory provides a way to bound the reliability of key inferences that underlie a family of decision tasks independent of particular data sets. For our task the critical decision variable is a binary variable *y* denoting the trajectory side, which is predicted based on trajectory information *x* (including motion and color) using a model with parameters *θ*. The basic parameter learning problem is to find the parameters that maximize the predictive probability of ${\hat{y}}_{t}$ at trial *t*:

3 $$p({\hat{y}}_{t}|{x}_{t},\theta )$$

In Bayesian approaches to active learning the objective is to request feedback for inputs for which the reduction in posterior entropy given feedback exceeds the cost of the request:

4 $$(H[0|{{\mathscr{D}}}_{t-1}]-{H}_{p({y}_{t}|{x}_{t},{{\mathscr{D}}}_{t-1})}[\theta |{y}_{t},{x}_{t},{{\mathscr{D}}}_{t-1}]) > c$$

Houlsby *et al*. (2011) show that the active learning criterion can be rewritten in terms of output entropies in an illuminating and computable form:

5 $${I}_{t}({y}_{t}|{x}_{t},{{\mathscr{D}}}_{t-1})=H[{y}_{t}|{x}_{t},{{\mathscr{D}}}_{t-1}]-{{\mathbb{E}}}_{p(\theta |{{\mathscr{D}}}_{t-1})}[H[{y}_{t}|{x}_{t},\theta ]]$$

This equation gives the parameter learning information gain for trial *t* based on the history of feedback data up to the previous trial ${{\mathscr{D}}}_{t-1}$. The equation has a useful interpretation as the increasing desirability of infoseeking with the uncertainty in the outcome prediction (first term), which we call the *per-item infogain*. The second term incentivizes precision in the parameters.

For parameter learning systems with perfect memory, both the left and right hand terms exponentially asymptote to the same constant value, rapidly reducing the incentive for additional information. In particular, when the posterior on the parameter collapses to a delta function, the parameter learning information gain converges to zero. In addition the two terms are related via Jensen’s inequality, which states that for concave functions like the entropy, the expectation of a function is always less than the function applied to the expectation. Because the marginal probability of the outcome given input and data is the expectation of $p({\hat{y}}_{t}|{x}_{t},\theta )$ over the parameter posterior, Jensen’s inequality means that the parameter learning information gain is always positive. Thus we expect parameter learning systems to show a decreasing incentive for feedback, with the desire for feedback to saturate *unless the uncertainty on the parameters stays high, due to parameter drift or forgetting*. In this case we should see a sustained information seeking, but this information seeking should depend critically on recent history. We make these predictions more precise in the section on parameter drift and information incentives below.

#### Information incentives for episodic learning

While parameter learning seems a natural way to learn, it requires knowledge of how to effectively parameterize a relationship. Storing information in parameters is space-efficient but doesn’t allow later testing or retesting of the initial parametric assumptions. At the other extreme, the brain could store all of the incoming data. That is highly space inefficient and unnecessary – typical events are predictable and don’t need to be stored. A principled compromise is to store events with high *surprisal*, events which are atypical and could yield alternative descriptions of the environment. Given a sequence of inputs *x*_*t*_ the system has learned to extract a sequence of observable predictors *y*_*t*_ (e.g. left-right for the asteroids game) from these stimuli producing an episodic history:

6 $${{\mathscr{D}}}_{t}=\{({x}_{1},{y}_{1}),\,\cdots ,\,({x}_{t},{y}_{t})\}$$

Given a new stimulus *x*_*t*+1_ the surprisal for a new feature value *y*_*t*+1_ is the entropy of the conditional distribution, which is also the *episodic infogain*:

7 $$Surprisal({y}_{t+1})=H[p({y}_{t+1}|{x}_{t+1},{{\mathscr{D}}}_{t})]$$

A rational sampling plan is to request feedback proportional to surprisal, which provides an episodic record of the environment in proportion to its unpredictability. When the predictive information is bounded by a sufficient statistic, *sampling according to surprisal will generate an apparently irrational behavior that collects information long after the sufficient statistic could have been characterized*. The value of this additional information is in providing a compact record of the environmental statistics. This record is **not** equivalent to translating the stored information into learning in the sense of improved performance, change in behavior, or a specific model of the environment. However, it is also clear that having stored information, the brain can internally analyze this data to aid in discovery, task transfer, or tracking.

Notice that investing in an intelligent environmental sampling method is only warranted when the storage capacity is constrained. Individuals with very high storage capacity may want to oversample the environment, while those with very low capacity may not have the luxury of sampling at all. The fact that storage costs control the sampling rate can produce large individual differences in behavior from an identical underlying strategy.

#### Parametric Learning with assumptions of volatility/forgetting

If information gain is computed under the assumption of volatility of the environment, then parameter learning can result in asymptotic ongoing information seeking. Parameter learning with volatility introduces a time series on base parameters *θ*_1:*T*_ = {*θ*_1_, …, *θ*_*T*_} with hierarchical parameters *η* that represent the rate of change of the environment. These models all share the property that information stored in the base-level parameters is focused on a restricted range of the environmental history, a phenomenon equivalent to a type of forgetting. Information incentives under forgetting share key qualitative features that we derive below.

Sequential Bayesian inference provides a framework for specifying the optimal sequential inference for base parameters and hierarchical parameters. The posterior on the base parameters can be written in a general “filtering” update form which aids in thinking about the information incentive.

8 $$P({\theta }_{T}|{{\mathscr{D}}}_{T},\eta )=\frac{P({Y}_{T}|{X}_{T},{\theta }_{T})P({\theta }_{T}|{\theta }_{T-1},\eta )P({\theta }_{T-1}|{{\mathscr{D}}}_{T-1},\eta )}{P({Y}_{T}|{X}_{T},{{\mathscr{D}}}_{T-1})}$$

Focusing on the new information provided by a sample means comparing the entropy of the posterior before and after updates,

9 $$IG({y}_{T})=H({\theta }_{T}|{{\mathscr{D}}}_{T},\eta )-H({\theta }_{T-1}|{{\mathscr{D}}}_{T-1},\eta )$$

Now

10 $$\begin{array}{rcl}H({\theta }_{T}|{{\mathscr{D}}}_{T},\eta ) & = & H({y}_{T}|{\theta }_{T})+H({\theta }_{T}|{{\mathscr{D}}}_{T-1})-H({y}_{T}|{{\mathscr{D}}}_{T-1})\\  & = & H({y}_{T}|{\theta }_{T})+\{{\rm{\Delta }}({S}_{T},{\rho }_{\theta \to \theta \text{'}})+H({\theta }_{T-1}|{{\mathscr{D}}}_{T-1})\}-H({y}_{T}|{{\mathscr{D}}}_{T-1})\end{array}$$

So that the information gain simplifies into three interpretable terms:

11 $$I{G}_{T}={U}_{T}(\theta )-{S}_{T}({y}_{T})+{{\rm{\Delta }}}_{T}$$

where *U* is the uncertainty in outcome *y* given the parameter *H*(*y*_*T*_|*θ*_*T*_), *S*_*T*_ is the surprisal, and Delta is a new term which quantifies the increase in uncertainty due to the assumption of volatility. Delta exactly quantifies the increase in uncertainty from the prediction under volatility, which we represent by a Markov transition of the parameter,

12 $$P({\theta }_{T}|{{\mathscr{D}}}_{T-1})=\int P({\theta }_{T}|{\theta }_{T-1},\eta )P({\theta }_{T-1}|{{\mathscr{D}}}_{T-1})d{\theta }_{T-1}$$

This term can be well approximated whenever the volatility process has a stationary distribution. Let *ρ*_*θ*→*θ*′_ represent the stationary distribution associated with the Markov Chain of the volatility process ${\rho }_{\theta \to \theta \text{'}}={\int }^{}P(\theta \text{'}|\theta ){\rho }_{\theta \to \theta \text{'}}\,d\theta$ where the *P*(*θ*′|*θ*) = *P*(*θ*′|*θ*, *η*) is the process that governs changes in the parameter between trials and the volatility parameter *η* controls the rate of diffusion.

For notational convenience, we represent the parameter posterior using the notation ${b}_{T,H}=P({\theta }_{T}|{{\mathscr{D}}}_{T-H})$ which indicates the parameter posterior at trial *T*, given a history of feedback where the last feedback occurred *H* trials ago. Using Donsker-Varadhan large deviation theory for stationary Markov Processes [Donsker Varadhan 1978], the volatility transition pulls the posterior toward the stationary distribution at an exponential rate given by the KL divergence between them *KL*(*ρ*_*θ*→*θ*′_∥*b*_*t*,0_). This means that the belief can be well approximated as a convex combination of the last belief update from feedback and the *H* transitions under the volatility process:

13 $${b}_{T,H}=(1-{e}^{-H\cdot KL({\rho }_{\theta \to \theta ^{\prime} }\parallel {b}_{T-H,0})}){\rho }_{\theta \to \theta ^{\prime} }+{e}^{-H\cdot KL({\rho }_{\theta \to \theta ^{\prime} }\parallel {b}_{T-H,0})}{b}_{T-H,0}$$

Because our volatility parameter controls the rate of diffusion, we can identify it as the KL itself, allowing us to simplify the above result as

14 $${b}_{T,H}=\rho +{e}^{-H\eta }({b}_{T-H.0}-\rho )$$

Using the constant *k* to denote the entropy of the stationary distribution *ρ*, the entropy change after *H* no feedback trials is then approximately

15 $${{\rm{\Delta }}}_{T,H}=k\cdot (1-{e}^{-H\eta })$$

Which means the dynamic information gain for parameter learning increases rapidly at a rate determined by the volatility assumed about the environment. After parameter convergence, parametric information gain is dominated by this term. While the Delta term rapidly approaches a constant, the volatility term introduces two essential characteristics of dynamic parametric information gain immediately after feedback:

1. feedback should suppress information incentive on average
2. hazard function for additional feedback will increase with time elapsed since last feedback

These effects should occur after *every* feedback, which allows us to qualitatively test this class of information incentive models.

#### Local influence of feedback on uncertainty

During learning, before the parameter posterior has converged, whether feedback is positive or negative will also impact uncertainty estimates and information incentives. The impact of a data point on an estimate can be quantified by computing the influence function. Influence functions for entropy quantify how much the uncertainty estimates are impacted by a positive (success) or negative (failure) feedback. The idea is to assess how much the parameter estimate $\hat{\theta }$ would change if we allowed a feedback point *f* to perturb the estimate by ${\epsilon }$. Koh and Liang^47^ show that the influence of a data point on functions of the parameter like the entropy can be assessed through the formula:

16 $$Influence(f)=-\,{\nabla }_{\theta }H(\theta |{{\mathscr{D}}}_{T},{f}_{T})J{({\hat{\theta }}_{T})}^{-1}{\nabla }_{\theta }H(\theta |{{\mathscr{D}}}_{T},{f}_{T})$$

where *J* is the hessian of the entropy with respect to theta. In general, the effect of feedback depends on the value of the parameter. When *θ* is the Bernoulli performance parameter, positive feedback *increases* uncertainty for low performance and reduces uncertainty for high performance. This predicts a transient impact of feedback on information seeking during learning where

1. Positive feedback increases information incentive for performance less than 0.75
2. Negative feedback increases information incentive for performance greater than 0.75

#### Individual differences as multi-objective information sampling

Rather than assuming that information seeking is driven entirely by parameter learning or entirely by episodic incentive, we propose that information incentives are driven by multiple distinct causes. We consider parameter learning, episodic sampling and trait-level incentives plausible and distinct objectives, producing an overall information incentive *IG*_*total*_ with parameters that can vary between participants *s*

17 $$I{G}_{total}={K}_{s}+{\alpha }_{s}I{G}_{episodic}+{\beta }_{s}I{G}_{\theta }$$

where *K* is a trait-level desire for information, and the other two terms incentivize episodic sampling and parametric learning.

#### Information sampling for binary decisions

We can specialize the information gain equations to our experiment to generate more detailed quantitative predictions that remain fully general. In the information gain equations, the parameters represent anything that allows prediction of the output variables from the input variables, from the weights of a recurrent neural network to a simple bernoulli probability.

Whatever the parameterization, requesting feedback only provides information about these parameters through the output variable *y*. Because the trials in our experiment are drawn i.i.d., the sufficient statistic (information bottleneck) for *y* is the Bernoulli probability of left-right *θ*, and any parameters in our subject’s internal model will mediate their influence through this statistic. By the data processing inequality, the information provided by feedback to model parameters is bounded by the information available in the sufficient statistic. In other words, if ***w*** is a vector of higher level parameters, then the mutual information between ***w*** and *y* is less the the mutual information between the sufficient statistic and *y*, *I*(***w***; *y*) ≤ *I*(*θ*; *y*). This means that we can focus on the sufficient statistic *θ* and treat any other parameters as a function of this variable, e.g. $P(y|\theta (\overrightarrow{{\boldsymbol{w}}}),x)$. Thus the information incentive for ***w*** can be studied through *θ*, allowing us to make strong quantitative predictions.

The sufficient statistic for feedback is the bernoulli probability of the trajectory direction on trial *t* is given the trajectory information *x*. Because we provided clear color cues to the trajectory type, we simplify discussion by assuming each trajectory has its own parameters, indexed by a discrete random variable *x*_*t*_ and use the particular value *x* to denote which of these trajectories is observed on trial *t*. The feedback data for each trajectory type *x* are the counts of feedback for left and right observed at trial *t*: ${{\mathscr{D}}}_{t,x}=\{{n}_{t,x},{m}_{t,x}\}$, where *n* and *m* are the counts of how many times the trajectory is observed right and left respectively. The predicted feedback *y*_*t*_ has probability:

18 $$P({y}_{t,x}|{\theta }_{t,x},{{\mathscr{D}}}_{t,x})=P({y}_{t,x}|{\theta }_{t,x})={\theta }_{t,x}$$

and this probability has a confidence given by

19 $$P({\theta }_{x,t}|{{\mathscr{D}}}_{x,t})=\frac{1}{z}\{{{\theta }_{x,t}}^{{n}_{t}}{(1-{\theta }_{x,t})}^{{m}_{t}}\}P(\theta )$$

where *z* is a the normalization constant and *P*(*θ*) is the prior. Because rates of convergence do not critically depend on the prior, we assume a non-informative prior below. However, explicit priors can be incorporated to account for individual differences in initial information seeking and responses to feedback. Because the effectiveness of the prior saturates quickly with feedback, the prior is irrelevant to explaining sustained sampling behavior. Finally, to simplify notation, we sometimes suppress the trajectory index *x*.

Explicit forms of information gains based on this experimental model are given below.

#### Information gain for parameter learning

The bottleneck for parameter learning is the uncertainty of the posterior distribution of the sufficient statistic *θ*_*t*_:

20 $$H({\theta }_{t}|{{\mathscr{D}}}_{t})=B({\theta }_{t}t,(1-{\theta }_{t})t)+(t-2)\psi (t)-(t-1)\psi ({\theta }_{t}t)-(t-1)\psi ((1-{\theta }_{t})t)$$

where *ψ*(*x*) is the digamma function and *B*(*x*, *y*) is the beta function and we assume that the equivalent number of samples provided by the observed data is approximately *t*, and excluded a constant term due to the prior. The entropy above is a differential entropy expressing the log(volume) of the posterior on the unit interval. Using the recursion properties of the Beta and digamma functions for integer inputs, we can show that the expected change in entropy after an update is an inverse function of experience, rapidly converging to zero at an approximate rate:

21 $$r({\theta }_{t}|feedback)=\{\begin{array}{ll}\frac{\gamma }{(1-{\theta }_{t})}-2 & \,if\,feedback=success\\ \frac{\gamma }{{\theta }_{t}}-2 & \,if\,feedback=failure\end{array}$$

where *γ* ≈ 0.5774 is the Euler-Mascheroni constant.

Thus the expected rate is *α*(*θ*_*t*_) = *p*(*f* = 1|*θ*_*t*_)*r*(*θ*_*t*_|*f* = 1) + *p*(*f* = 0|*θ*_*t*_)*r*(*θ*_*t*_|*f* = 0), where *f* = 1 and *f* = 0 indicate the predicted feedback is correct or incorrect, respectively. Because the predicted feedback probabilities are the Bernoulli parameter: *p*(*f* = 1|*θ*) = *θ* and *p*(*f* = 0|*θ*) = 1 − *θ*, the expected rate simplifies to:

22 $$\alpha ({\theta }_{t})=-\,2+\gamma (\frac{{{\theta }_{t}}^{2}+{(1-{\theta }_{t})}^{2}}{(1-{\theta }_{t}){\theta }_{t}})$$

that is a convex function the probability, with the slowest rate when performance is at chance. This gives a final approximate form for the parameter learning information gain:

23 $$I{G}_{\theta }(t)\approx \frac{\alpha ({\theta }_{t})}{t}$$

This form provides simple qualitative predictions, both for information incentives based on predicted information, as well as the expected behavior of a parameter learning system after feedback - entropy increases after errors, and the more confident the system, the more an error incentivizes additional feedback.

#### Episodic information gain

Episodic information gain is the predictted value of feedback for identifying the outcome variable *y*_*t* + 1_, which is the expected reduction in entropy of *y*_*t*+1_ from feedback at trial *t* + 1 over the entropy of the prediction based on information available from the previous *t* trials. Because the expected entropy of after feedback *E*_*y*_[*H*(*y*_*t*+1_|*f*)] is zero if the feedback is perfectly encoded, we can approximate the information gain *IG* = *H*(*y*_*t*+1_|*θ*_*x*,*t*_) − *E*_*y*_[*H*(*y*_*t*+1_|*f*)] as:

24 $$I{G}_{episodic}(f|{{\mathscr{D}}}_{x,t})=H({y}_{t+1}|{\theta }_{x,t})={\theta }_{x,t}\,\mathrm{log}({\theta }_{x,t})+(1-{\theta }_{x,t})\mathrm{log}(1-{\theta }_{x,t})$$

This expression is the well-known self-information of a binary random variable, a concave U-shaped function of the sufficient statistic. Critically, this quantity only depends on past observations through a bottleneck represented by the parameter, because it is a sufficient statistic for our experiment.

### Computation of episodic information gain

We used the subject’s average proportion of correct steering decisions *μ*_*x*,*t*_ up to trial *t* for trajectory *x* as that participants’ perception of difficulty *θ*_*t*,*x*_, which provides an empirical estimate of the subject’s episodic information incentive as:

25 $${\widehat{IG}}_{episodic}(t)={\mu }_{t,x}\,\mathrm{log}({\mu }_{t,x})+(1-{\mu }_{t,x})\mathrm{log}({\mu }_{t,x})$$

Because this estimate is approximately a constant for the last 85% of the experiment, we used the value for *t* = *T*, the value at the end of the experiment for our aggregate predictions. We regressed this value against the proportion of feedback choices across the ten different difficulty levels. This produces ten proportions of feedback choices for each participant and allows for a linear regression test of the proportion of feedback choices as a function of information gains. This linear regression analyses can then be conducted in the aggregate (i.e., across participants) as well as at the individual participant level. We found that basing the episodic information gain on each subject’s observed feedback added variability without improving or modifying our predictions.

## Supplementary information

Supplementary information

## Data Availability

The data and analysis script files are available online via the *Open*. *Science Framework* public project entitled “Asteroid avoidance”.
