# Supplementary material for: Episodic curiosity for avoiding asteroids: Per-trial information gain for choice outcomes drive information seeking
Source: Sci Rep. 2019 Aug 2;9:11265. doi: 10.1038/s41598-019-47671-x (PMC6677824; doi:10.1038/s41598-019-47671-x)
Supplement: Supplementary file 1 — Supplementary information [file 41598_2019_47671_MOESM1_ESM.pdf]

SUPPLEMENTARY INFORMATION

Episodic curiosity for avoiding asteroids: Per-trial information gain for choice outcomes drive information seeking

Linus Holm[1*], Gustaf Wadenholt[1], Paul Schrater[2,3]

1. Department of Psychology, Umeå University, S-901 87 Umeå, Sweden
2. Department of Psychology, University of Minnesota, Minneapolis, MN 55455, USA
3. Department of Computer & Engineering Science, University of Minnesota, Minneapolis, MN 55455, USA
4. *Corresponding author: linus.holm@umu.se
5. Phone: +46 90 786 76 07

Additional linear mixed-model results

The relationship between intercept and information-gain parameters across participants (random by-subject effects) from the linear mixed-model on the information-gain and feedback request analysis reported in the main article, is displayed in Figure S1. As seen in Fig S1, a few participants almost always request feedback, leaving little opportunity for an information gain slope.

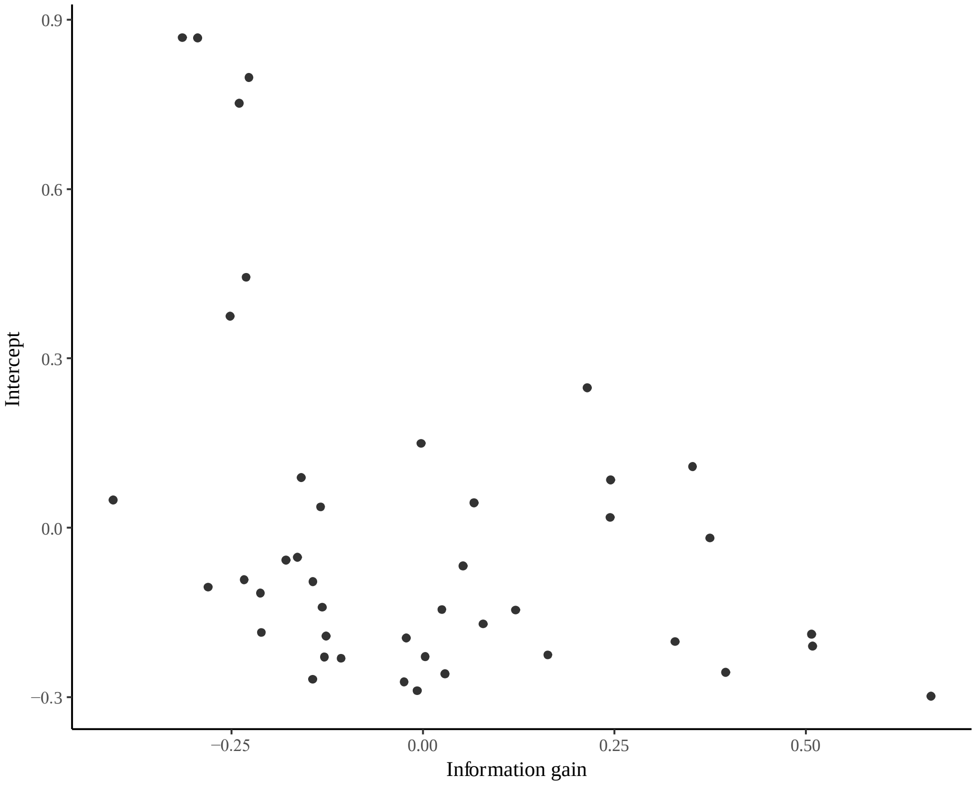

Figure S1. Scatterplot of random by-subject effects from the linear mixed effects model.

Effect of time in test on information seeking. Figure S2 displays the intercepts and slopes of the information gain - feedback request-rate relations for each of the eight blocks of the test. As seen, there is rather little change in the association across blocks (i.e., time in the test); the slopes are pretty similar. Consistently, we find no reliable effect of block on the association between information gain and feedback request (β = .00, p > .05) as summarized in table S3.

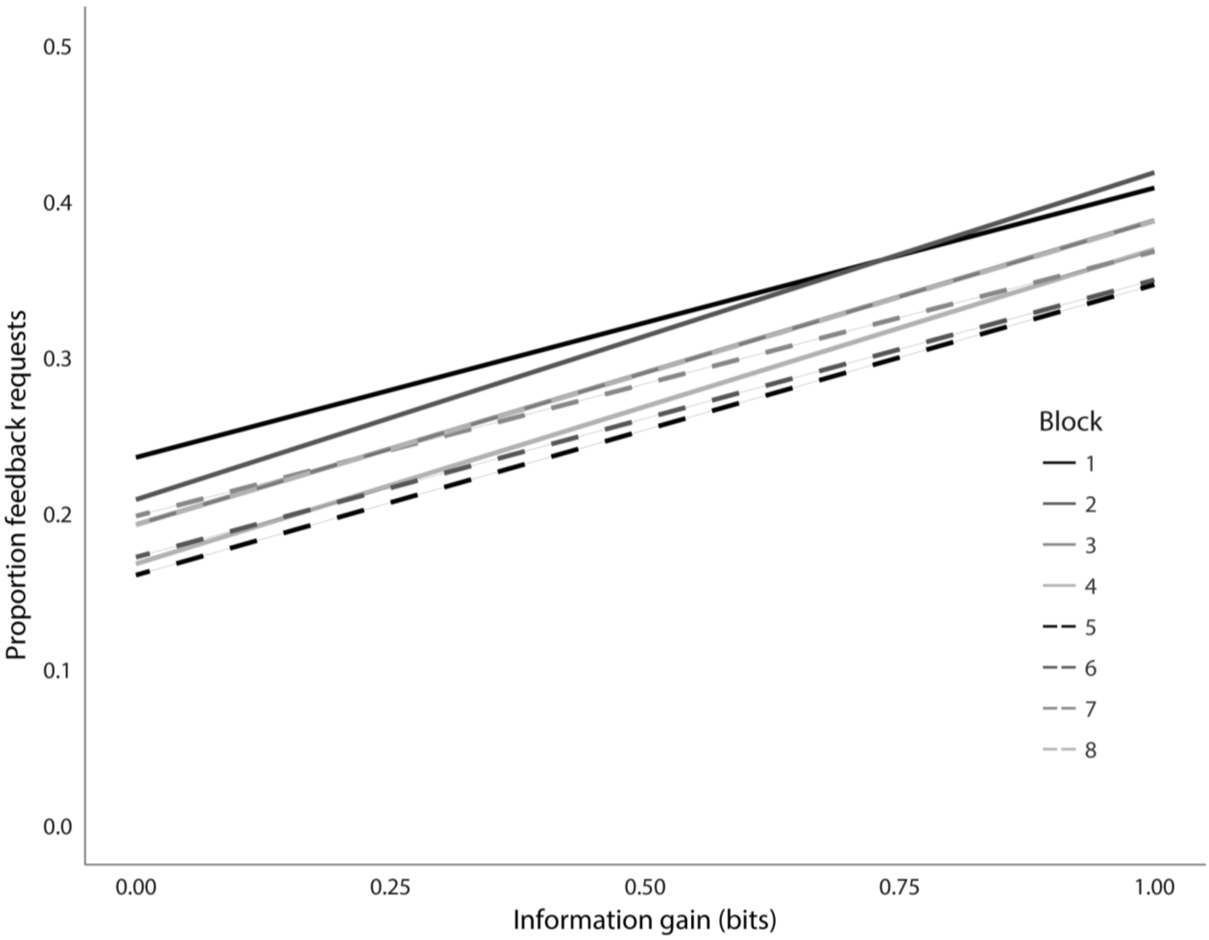

Figure S2. Fixed effects of eight separate linear mixed effect analysis for information gain on feedback requests, for each block of 100 trials (10 trials of every trajectory). Intercept range from .17 to .24 (all *p*s <.05), and Information gain coefficient range from .17 to .21 (all *p*s < .001).

Table S1

Results from the linear mixed-effects analysis of Information gain and Information gain by Block on Feedback choice

**Formula**	$FB \sim IG\ 0 + Block + IG \times$ *Block + (IG + Block + IG × Block|S)*

Fixed effects	*β* (95% CI)	*t* (df)
Intercept	.24 (.13 – .34)	4.28*** (41.43)
Information gain	.17 (.09 – .25)	4.28*** (39.61)
Block	-.00 (-.02 – .01)	-.70 (33.56)
Information gain × Block	-.00 (-.01 – .01)	-.34 (27.78)
**Random effects**		
σ^2	.02	
[illegible]$_{00, Subject}$	.12	
ρ_{01}	-.40	
$ICC_{Subject}$	.83	

Notes. All *dfs* subject to Satterthwaite corrections. *p < .05. **p < .01. ***p < .001.

σ^2 - within-group variance

[illegible]$_{00, Subject}$ - between-group-variance

ρ_{01} - random-slope-intercept-correlation

$ICC_{Subject}$ - intraclass correlation

$N_{subjects}$ = 43.

Observations = 3440.

Individual differences in information seeking and performance

Participants differed substantially in information seeking rates. To get a sense of its relationship with other variables, we compared information seeking rates with information gain and steering reaction times (Fig S3). As seen in Fig S3, there appears to be no obvious relationship between overall feedback request rate and information gain, or between reaction time and feedback request rate and infogain, respectively.

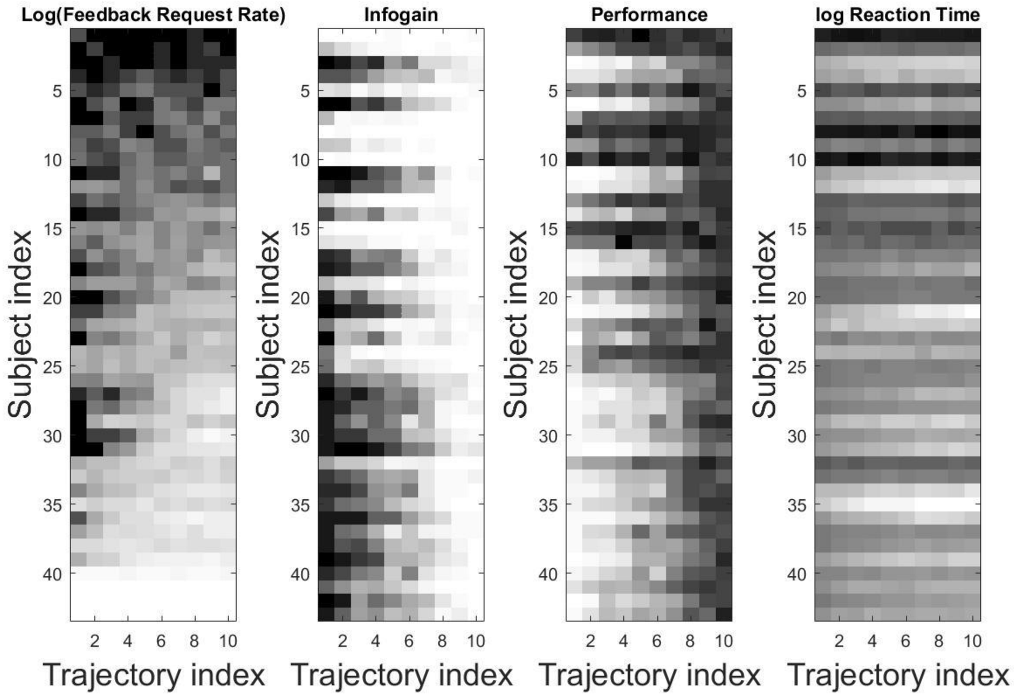

Figure S3. Summary of participants feedback request rates, expected information gains, performance and reaction times as a function of task difficulty (trajectory), respectively. All panels sorted on subject (i.e., top row refers to the same subject across all four panels). Lighter color reflects higher value on each respective quantity.

An alternative way of analyzing information seeking behavior to the episodic curiosity method described in the main paper is to test if participants selected feedback with an aim of maximizing information gain. If so, then given a certain willingness to wait, the requests should be spent on the most informative (i.e., difficult) trajectories. This allowed us to express each subject's feedback selections as a fraction of an ideal observer for the corresponding rate of feedback requests.

Moreover, to distinguish choice from chance level, we reset the proportion based on the range of possible information gains for each participant. The participant distribution of these normalized ideal information-gains are displayed in Figure S4. As seen in Figure S4, only 4 participants select less than 0.5 of ideal information-gain, and the remainder of the participants are clearly biased towards an ideal level of info-gain given feedback rate.

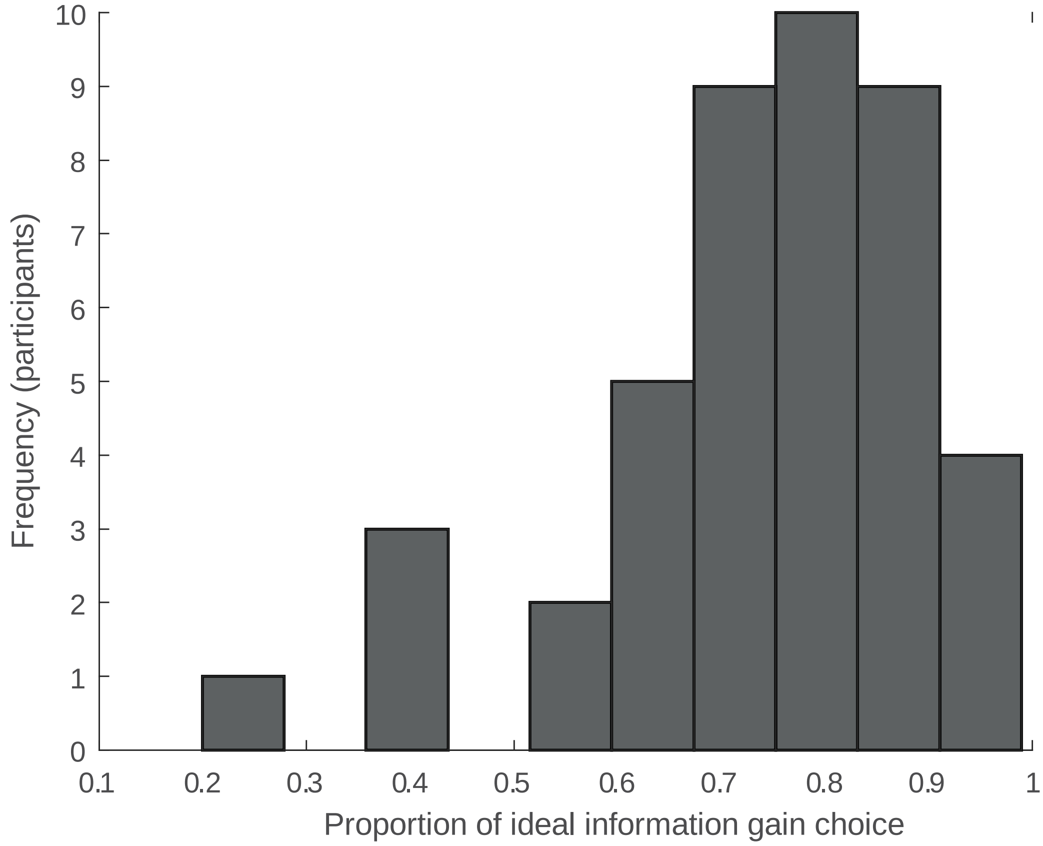

Figure S4. Participant distribution of normalized fraction of ideal info-gains.

To test how much overall variance infogain accounted for in our data, we performed a PCA on feedback requests across difficulty (trajectory) and participants. As seen in Fig S5, the first

component accounts for about 98% of the variance and corresponds with participant overall

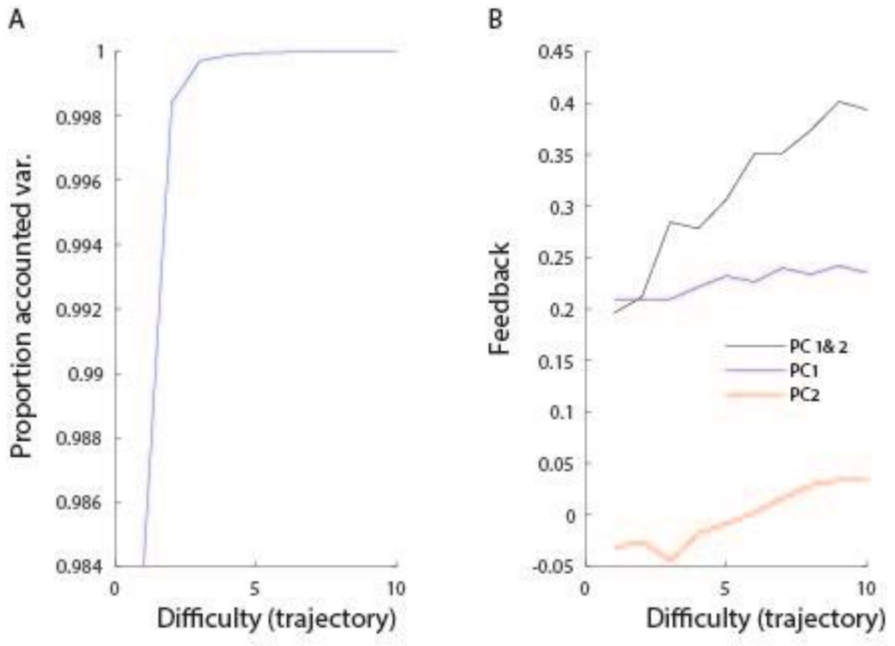

feedback rate.

Figure S5. Output of PCA based on feedback request rates and difficulty. Panel A displays proportion of variance accounted for as a function of factor. Panel B indicates factor average as a function of difficulty.

Eye movements

Participants requested feedback by fixating the cockpit area on the screen with their eyes. It is possible that this procedure guided participants' eye movements differently for different trajectories. To investigate this possibility, we aggregated all eye fixation positions from all participants for each of the ten trajectories, respectively. Three of these aggregated sets are displayed in Figure S6.

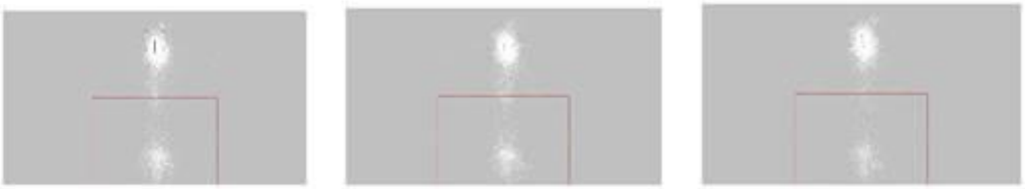

Figure S6. Aggregated fixations from all participants in trajectories of 0.005, 0.027 and 0.18 radians from the midline (panels from left to right, respectively). Red dotted line indicates zone of feedback request. Black lines indicate trajectory angle.

As seen in Fig S6, there was no substantial difference in fixation positions between the easiest and most difficult trajectories. Importantly, fixations seem not to have extrapolated the easiest trajectory (rightmost panel in Fig S6) outside of the feedback request box. Thus, there appears to be no confound of trajectory with accidentally driving fixations away from the feedback zone. In other words, extrapolating from the trajectories would bring the eyes within the feedback zone for all trajectories. Note that there seems to be a decreasing density of fixations in the feedback zone moving from the left to rightmost panel indicating more frequently requested feedback, the more difficult the avoidance decision was.
